# Notes and News

**Published:** 1890-01-18

**Authors:** 


### NOTES AND NEWS.

COLLECTED AND COMMUNICATED.

IN the hospital of Jefferson College, Philadelphia, a very skilful operation was lately performed upon a child about six years old, who was suffering from epilepsy. The skull was opened, and the affected part of the brain stretched. The operation proved successful, and the child is progressing favourably. In America the case is attracting great attention.

AT the monthly meeting of the committee of Manchester Royal Infirmary a letter was read from Mr. Matheson proposing that a site for the new hospital of St. Mary should be found in the infirmary grounds. The subject was deferred. A vote of condolence with Lady Heron on the death of her husband was passed. Sir Joseph Heron was ever a good friend to the infirmary.

A HINDOO has lately been giving some particulars of the different deities of the Chinese, and the Heathen Chinee seems to have a special delight in worshipping disease. A miserable, broken down-looking figure is known to everybody as "His Excellency the Asthma," who crouches in close proximity to the writhing "Mr. Muscle-and-Bone-Pain." It would be too much to have a separate god for every single disease that flesh is heir to, so the ingenious Chinaman has devised a useful deity to cover all kinds of ailments, calling him "Mr. Imperfect-in-Every-Part-of-his-Body." The idol is very repulsive in appearance, having sore eyes and a hare-lip, an ulcer here, a diseased bone there. To him a sufferer from any disease, unprovided with a god of its own, offers a symbol of the part affected, cut out in gilt paper or silk, and he goes away with the assurance that he will be cured.

THE report of the Devonshire Hospital and Buxton Bath Charity shows that the management of hospital patients is not always easy. Of the 2,431 in-patients admitted during the year ended December 31, 1889, the large number of 2,274 were discharged as much improved; only fifty-six as no better; twenty-four were discharged at their own request; six had to be sent away on account of drunkenness; six were found to be infested with vermin; three were ascertained to be unfit cases for the hospital; two were discharged for misconduct; five left without report; seven died; and forty-eight remained on the books at the end of the year. No fewer than seventeen serious surgical cases were treated during the year, but over 2,000 of the cases were rheumatism in different forms. The financial position of the charity is satisfactory, and we hope the committee will not try to reduce the cost per patient by limiting the food, as they say has been suggested to them.

THE first annual meeting of Coldstream Cottage Hospital subscribers was held in December. About 50 cases have been successfully treated during the year as appears from Dr. Henderson's report, and no death has yet occurred in the hospital. It has never been altogether empty; and as many as ten cases have been under treatment at the same time. Finances are satisfactory, and there is a balance of £57 in hand.

WE have received a serious remonstrance on the subject of the word competition which appears weekly in our pages, and we learn for the first time that it fosters waste of time and raises false hopes. Our only desire is to provide some amusement which nurses like, and as about fifty enter for the competition we take this as a proof that a fair proportion of our readers are in favour of the continuation of this competition. It is in far greater favour that the examination question competition, which may be more improving, but does not seem to be so suitable for off duty hours.

IT is delightful to hear that the fourth annual service in aid of the Dewsbury Infirmary took place in the Roman Catholic Church of St. Paulinus. Charity is the one bridge which covers the chasms between the different Christian creeds, and through charity these chasms are yearly growing less deep. The service commenced at three o'clock with the ordinary vespers, the Rev. P. Mulcahy, the pastor, officiating. He said that he had to thank the Mayor of Dewsbury and the members of the Corporation, also the president and members of the Infirmary Board, in his own name, and also in the name of his humble flock, for the honour they had done the Roman Catholic portion of the community by attending the service that afternoon. It was an honour he did not expect, it was an honour he could not repay, and it was an honour that would be fully appreciated by every member of his flock. He took the present opportunity of wishing them all a prosperous and a happy new year, and many returns of the day. The rev. gentleman then took his text from the fifteenth chapter of the Book of Deuteronomy, "For the poor shall never cease out of the land; therefore I command thee, saying, Thou shalt open thine hand wide unto thy brother, to thy poor, and to thy needy in thy land." The collection amounted to over £20.

AMONGST other items of news from New York, we learn that the Babies' Wards of the Post Graduate Hospital, which were opened for the first time this year, treated 100 babies during the summer. Nurses are specially trained here in the care of infants.—The trial of the "Rev." William H. Ramscar, charged with assaulting John Laverty, an inmate of his "home," with a club in September last, is going on. Ramscar is running the "National Unsectarian Home for Old Men" in One Hundred and Eighty-first-street, Washington Heights, and like many other private charities it seems an abode of cruelty.—Work was begun lately upon the new addition to the Roosevelt Hospital at Fifty-ninth-street and Ninth-avenue. A short time ago the hospital received 200,000dols., which had been left to it by a Mr. Sims, for the purpose of building and maintaining a new operating-room.—In the *Medical Record* of December 7th was published a letter from Dr. Robert H. M. Dawbarn, of No. 345, West Fifty-sixth-street, in which the present administration of the civil service boards was severely attacked. The doctor charged that, in appointments to civil service positions, merit and efficiency are made subservient to considerations of political policy. In substantiation of this charge he cited several cases that had come under his notice.—The deaths are announced of Dr. Seth Pancoast, the well-known homœopathist of Philadelphia, and Dr. Charles Henry Nichols, superintendent of the Bloomingdale Insane Asylum since 1877.

The following vacancies are announced this week, the amount of the salary in each case being given after the name of the institution :

**Resident Medical Officers.**

Hospital for Women, Soho-square. Clinical Assistants.
Belgrave Hospital for Children. House Surgeon.
Birmingham General Hospital. Two Assistant House Surgeons—Board, &c.
City Road Chest Hospital. House Physician—£50, board, &c.
Aylesbury Infirmary. Surgeon and Apothecary—£80, board, &c.
Fisherton Asylum, Salisbury. Assistant Medical Officer—£100, board, &c.

**Non-Resident Medical Officers.**

Bristol Eye Hospital. House Surgeon—£120.
Manchester Clinical Hospital for Women and Children. Aural Surgeon.
London Hospital Medical College. Curator's Assistant—£52.
St. Saviour's, Southwark. Medical Officer of Health—£200.
Parish of Whittesley. Medical Officer—£70.

THE magisterial investigation into the charge against Dr. Herman Tribe, of Chatham, of criminally assaulting Rose Jarvis came to a close on Monday. The magistrate, who had already struck out a charge of drugging, now said there was nothing to go before a jury. He discharged the prisoner, and said he did so with the concurrence of the Public Prosecutor.

AMELIA WOOLLENTINE, a young woman who described herself as a nurse, was tried at the South London Sessions this week for theft. According to the evidence, she had gained admission to charitable institutions, from which, a few days later, she decamped with property valued in one instance at £9, and in another at £18. Sir P. Edlin sentenced her to five years' penal servitude.

THE death of the Empress Augusta will be a great loss to many medical charities in Berlin, particularly to the ambulance department and the nursing institutions. The Empress was eminently a charitable and peaceable woman; she hated war, and she ever worked hard in the cause of the wounded soldiers who suffered for their country. She visited the hospitals constantly, and with her own hands ministered to those who were hurt in the Franco-German campaign. At Coblentz, where the Empress usually spent the summer, she was a perfect Providence to the people and led every charitable movement.

PROF. SIR DOUGLAS MACLAGAN took the chair at the annual meeting of the subscribers to the Edinburgh Royal Infirmary. The percentage of deaths to the whole of the ordinary medical and surgical cases treated was 6·4; deducting the deaths that occurred within 48 hours after admission, the percentage was reduced to 4·9. The number of patients treated in the Convalescent House during the year was 990, being 70 less than during the preceding year. During the past year the large number of 8,606 in-patients have been treated in the wards of the infirmary; while over 220 patients had, on an average, attended daily (Sundays excepted) in the waiting-rooms of the different departments, medical, surgical, and special. For the infirmary there had been received during the past year legacies and donations of £100 and upwards, amounting to £30,543 12s. 11d. This large sum had enabled the managers to reduce the building debt by £12,072 9s. 6d. The ordinary income for the year now reported on, which was £30,507 15s. 4d., as compared with £30,085 13s. 1d., showed an increase of £422 2s. 3d. The report was deemed satisfactory by those present.

THE visitors to the Evelina Hospital last week had an unexpected entertainment. Captain Shaw was present at the hospital as one of the committee, and he invited the Lord Mayor and others to go across the road to the headquarters of the Metropolitan Fire Brigade and see the engines turn out for an imaginary fire. It was most extraordinary to see the rapidity with which the horses were got in and the engines got away; within 25 seconds from the time the warning bell rang the engines were galloping down the street.

THE parish doctors have had a hard time of it during the influenza epidemic, so have the hospital physicians and nurses for that matter. A doctor in the North of London, who has a huge parish under his charge, got influenza himself, and never had time to recover properly from it, so many of his patients needed his services. You cannot attend to 100 extra patients a day without a serious strain on your resources, and few people seem to realise the extent of the epidemic. Yet every staff of workers, printers, postmen, and others, has been reduced about one-third, concerts have been postponed, and football teams have played five short each side. It is certainly the most widespread epidemic we have had for twenty years.

THE annual general meeting of the Royal Ear Hospital, Frith-street, Soho, was held in the hospital on Tuesday, the 14th inst., John Carr, Esq., J.P., treasurer, in the chair. The secretary reported that during the year just closed there were 8,420 out-patient attendances; that 102 patients had been admitted into the in-patient wards, and that these numbers were the largest on record since the foundation of the institution in 1816. The financial report showed that there had been an increase in the amount of the subscriptions and donations as compared with the previous year, but that larger subscriptions and donations are urgently needed to meet the ever-increasing demands on this old-established and most useful charity. A vote of thanks to the surgeons, treasurer, secretary, and matron brought the proceedings to a close.

AT the last meeting of the Metropolitan Asylums Board, Mr. Strong, the chairman of the "Exmouth" training ship committee, presented a special report in reference to the serious outbreak of influenza which had occurred on that vessel. Mr. Strong said the first outbreak occurred on Friday, the 3rd inst., when 33 lads were removed to the infirmary and three officers were attacked. The next day 53 lads and five officers were suffering from the complaint, while on the Sunday there were eight officers sick, and 92 boys in the infirmary and 40 on the orlop deck suffering from the influenza. On Monday, the 6th inst., 193 lads were laid up—103 on the orlop deck and 90 in the infirmary—while seven officers were sick; and on Tuesday no fewer than 225 lads were sick. The infirmary was then full, and 135 boys were laid up on the orlop deck. There was a drop on Wednesday, when 88 lads were under the doctor's hands, and the numbers decreased to 83 on Thursday and 81 on Friday. Mr. Strong added that none of the cases had been attended by serious results, and that the lads were cheerful under their ailments, and were rapidly recovering. The South-Eastern Hospital Committee reported the death, on December 31 last, of Mr. P. R. Ponsford, from diphtheria, contracted during the discharge of his duties as assistant medical officer. Upon this report a question arose as to whether protection masks for the mouth and nostrils, which the board had provided for its officers, had been used in this case, and it was asserted that they had not. The matter was referred to the committee for report.

